# Neuronal influences are necessary to produce mitochondrial co-localization with glutamate transporters in astrocytes

**DOI:** 10.1111/jnc.12759

**Published:** 2014-06-16

**Authors:** Christopher I Ugbode, Warren D Hirst, Marcus Rattray

**Affiliations:** * Reading School of Pharmacy, University of ReadingReading, UK; † Bradford School of Pharmacy, University of BradfordBradford, UK; ‡ Neurodegeneration and Neurologic Diseases, Pfizer Neuroscience Research UnitCambridge, Massachusetts, USA

**Keywords:** co-culture, EAAT2, Glial cell, glutamate transporter, rho kinase, TRAK2

## Abstract

Recent evidence suggests that the predominant astrocyte glutamate transporter, GLT-1/ Excitatory Amino Acid Transporter 2 (EAAT2) is associated with mitochondria. We used primary cultures of mouse astrocytes to assess co-localization of GLT-1 with mitochondria, and tested whether the interaction was dependent on neurons, actin polymerization or the kinesin adaptor, TRAK2. Mouse primary astrocytes were transfected with constructs expressing V5-tagged GLT-1, pDsRed1-Mito with and without dominant negative TRAK2. Astrocytes were visualized using confocal microscopy and co-localization was quantified using Volocity software. Image analysis of confocal z-stacks revealed no co-localization between mitochondria and GLT-1 in pure astrocyte cultures. Co-culture of astrocytes with primary mouse cortical neurons revealed more mitochondria in processes and a positive correlation between mitochondria and GLT-1. This co-localization was not further enhanced after neuronal depolarization induced by 1 h treatment with 15 mM K^+^. In pure astrocytes, a rho kinase inhibitor, Y27632 caused the distribution of mitochondria to astrocyte processes without enhancing GLT-1/mitochondrial co-localization, however, in co-cultures, Y27632 abolished mitochondrial:GLT-1 co-localization. Disrupting potential mitochondrial: kinesin interactions using dominant negative TRAK2 did not alter GLT-1 distribution or GLT-1: mitochondrial co-localization. We conclude that the association between GLT-1 and mitochondria is modest, is driven by synaptic activity and dependent on polymerized actin filaments.Mitochondria have limited co-localization with the glutamate transporter GLT-1 in primary astrocytes in culture. Few mitochondria are in the fine processes where GLT-1 is abundant. It is necessary to culture astrocytes with neurones to drive a significant level of co-localization, but co-localization is not further altered by depolarization, manipulating sodium ion gradients or Na/K ATPase activity.

The glutamate transporter GLT-1 [known as Excitatory Amino Acid Transporter 2 in humans] is an abundant astrocyte plasma membrane protein, with a critical role in maintaining L-glutamate homeostasis in the central nervous system, accounting for at least 95% of all glutamate uptake in the CNS (Danbolt 2001; Jiang and Amara 2011; Vandenberg and Ryan 2013). Astrocyte glutamate transporters limit glutamate receptor activation (Huang and Bergles 2004) and control glutamate synaptic transmission, particularly under conditions of high-frequency stimulation of neurons (Beart and O'Shea 2007; Beurrier *et al.* 2009). Activity of astrocyte glutamate transporters controls the glutamate:glutamine cycle which is required for neurons to maintain adequate levels of glutamate for transmission (Uwechue *et al.* 2012). Sodium ions which enter astrocytes during glutamate uptake are actively extruded, at the expense of ATP synthesized in mitochondria. A large portion of the brain's ATP turnover is required to remove glutamate from the synaptic cleft (Sibson *et al.* 1998; Anderson and Swanson 2000). Recent evidence suggests that glutamate transporters, localized in the plasma membrane, exist in protein complexes to facilitate this function, namely a direct protein: protein interaction with sodium-potassium ATPase (Rose *et al.* 2009) and interaction through undetermined partners with mitochondrial proteins (Genda *et al.* 2011). Therefore, an emerging theory is that GLT-1 is part of an activity-dependent macromolecular complex in astrocytes which efficiently couples energy provision to demand to maintain effective neurotransmission (Genda *et al.* 2011; Jackson *et al.* 2014).

This theory predicts that GLT1: mitochondrial co-localization at the plasma membrane of astrocytes should become elevated under conditions where demand is increased. Indeed, GLT-1 expression in astrocytes as determined by functional assays and protein expression, is dependent on neurons, or factors secreted from neurons (Swanson *et al.* 1997; Perego *et al.* 2000; Poitry-Yamate *et al.* 2002; Benediktsson *et al.* 2012), with deafferentation or loss of neuronal activity leading to loss in glutamate transporter levels (Ginsberg *et al.* 1995; Ouyang *et al.* 2007). Recent evidence shows that GLT-1 distribution within astrocytes is highly dynamic, with GLT-1 localization in astrocyte processes (filopodia) increasing following activation of neurons (Benediktsson *et al.* 2012).

Mitochondria in astrocytes have been reported to be highly motile (Mason *et al.* 1988), with their activity-dependent mobility dependent on both interactions with microtubules and actin filaments (Kremneva *et al.* 2013). GLT-1:mitochondrial co-localization has been suggested to predominate in the fine processes (filopodia) of astrocytes (Genda *et al.* 2011). However, many filopodia have a small diameter which would be expected to exclude mitochondria (Hertz *et al.* 2007; Lavialle *et al.* 2011) and the protein machinery which controls mitochondrial dynamics in astrocyte filopodia remains unknown. The interaction may be dependent on actin, as rho kinase inhibition which causes remodelling of actin filaments, causes astrocyte stellation and increased cell surface Excitatory Amino Acid Transporter 2 (Lau *et al.* 2011). In neurons a family of kinesin adaptor proteins (TRAKs) have been specifically implicated in mitochondrial trafficking into fine dendritic processes, through their association with the transmembrane mitochondrial proteins including Miro1 (MacAskill *et al.* 2009), with knockdown of TRAK family members, particularly TRAK2, reducing mitochondrial transport in neurons (Misko *et al.* 2010; Brickley and Stephenson 2011; Lopez-Domenech *et al.* 2012; van Spronsen *et al.* 2013). It is notable that TRAK2 is also involved in the trafficking of a number of transmembrane ion channels and receptors including Kir2.1, and GABA_A_ receptors in neurons, and it is possible that this protein has a role in astrocytes.

In this study, we sought to define the extent of co-localization of GLT-1 with mitochondria in primary cultures of astrocytes. To test whether GLT-1 association with mitochondria was regulated we used two conditions, comparing pure astrocyte cultures with astrocytes co-cultured with neurons. Co-cultures were also stimulated with 15 mM potassium chloride for 1 h to drive neuronal excitability and challenged with compounds that modulate Na^+^ gradients and Na/K ATPase activity. In addition, we tested whether dominant negative TRAK2 could disrupt potential GLT-1: mitochondrial associations.

## Materials and methods

### Animal groups

Timed mated female NIH Swiss mice (Harlan, UK) were maintained and used according to the UK Animals (Scientific Procedures) Act (1986) and local ethical guidelines. Animals were killed using cervical dislocation and cerebral cortices from E15 mouse embryos were obtained and cells were mechanically disassociated by triturating (20 times) with a sterile fire polished 230 mm glass Pasteur pipette (VWR International, East Grinstead, UK) pre-coated with sterile foetal bovine serum [Foetal bovine serum (FBS), Biosera, East Sussex, UK] in phosphate-buffered saline (PBS, Ca^2+^ and Mg^2+^ free) supplemented with glucose (33 mM), penicillin (100 units/mL) and streptomycin (0.1 mg/mL) as previously described (Hoppe *et al.* 2013). The cell suspension was centrifuged (5 min, 200 *g*) and re-suspended in the appropriate media. For astrocyte cultures, cells were plated in astrocyte specific media (DMEM/F-12 (HAM), Life Technologies, Grand Island, NY, USA) supplemented with glucose (33 mM), L-glutamine (2 mM), FBS (10%) and sodium bicarbonate (13 mM) at a density of 4 × 10^5^ cells per well, into 12 well plates containing 13 mm glass coverslips pre-coated with poly-L-ornithine hydrobromide (1.5 μg/mL) for 24 h. After 10 days *in vitro* (DIV), primary cortical astrocytes were 80–90% confluent. Neurons were removed by banging the plates followed by a wash with PBS and the addition of fresh media. Cultures were transfected at day 11 and used at day 14.

Astrocyte-neuron co-cultures were plated in Eagle's Minimum Essential Medium with Earle's balanced salt solution without L-glutamine (MEM Eagle with Earles balanced salt solution, Lonza, Slough, UK) supplemented with L-glutamine (0.5 mM), glucose (15 mM), gentamicin sulphate (10 mg/mL) and 5% heat inactivated horse serum (Sigma-Aldrich, St. Louis, MO, USA). Cells were seeded at a density of 4 × 10^5^ cells per well, into 12 well plates containing 13 mm glass coverslips which had been pre-coated with poly-D-lysine hydrobromide (0.1 mg/mL) for 10 min, air dried, then coated with FBS. Astrocyte cultures showed 95% purity and astrocyte – neuron co-cultures had 60% astrocytes to 40% neurons as assessed by immunostaining for glial fibrillary acidic protein (GFAP) (M076101-2, Dako UK Ltd., Ely, UK) and microtubule-associated protein 2 (MAP-2) (SMI-52R, Covance Inc., Leeds, UK), (data not shown). These cultures were transfected at day 14 and used at day 17, the time was chosen to optimize the electrophysiological activity of the neurons (results not shown).

#### Rho kinase inhibition and astrocyte toxicity

Primary mouse astrocytes (11 DIV) were treated with saline (Vehicle – 0.1% total volume) and varying concentrations of Y27632 (1, 10 and 100 μM) in 12 well plates and left for 24 h. After treatment, cells were washed with hank's buffered media (HBM) buffer (4.76 g HEPES, 40.88 g NaCl, 0.372 g KCl, 0.42 g NaHCO_3_, 0.1654 g NaH_2_PO_4_, 0.3 g Glucose and 240 μL of 0.5 M CaCl_2_ (Sigma, St. Louis, MO, USA)) and incubated with 500 μL of MTT (3-(4,5-dimethylthiazol-2-yl)-2,5-diphenyltetrazolium bromide) buffer [0.5 mg/mL Thiazoyl Blue Tetrazolium Bromide (Sigma) in HBM] at 37°C for 1 h. The formazan precipitate was then solubilized with 300 μL dimethylsulfoxide per well. 200 μL from each well was transferred to a 96 well plate and absorbance was measured using a plate reader (Flexstation 3, Molecular Devices. λ = 490 nm).

#### TRAK2 Expression in primary mouse astrocytes

RNA was extracted using the re-agent RNA Bee (AMS Biotechnology, Abingdon, UK). All RNA samples were derived from cells growing in six well plates (8 × 10^5^ cells per well). RNA bee was added to each well (1 mL) and left on ice for 5 min. RNA was then isolated using the chloroform based method of extraction, in accordance with the manufacturer's protocol. RNA concentrations were calculated using a NanoDrop 2000 spectrophotometer (Thermo Scientific, Waltham, MA, USA). A high capacity cDNA kit (4368814 – Life Technologies) was used to transcribe 2 μg of RNA. To perform PCR, the cDNA reaction mixture was diluted 1 : 20 in Tris-EDTA buffer (Sigma) and 3 μL of cDNA was combined with 2 μL of both the forward and reverse primers (2.5 μM stock, final concentration 100 nM) and 43 μL of Megamix pcr master mix (Microzone) to create a 50 μL reaction volume per well. Each cDNA sample underwent 30 cycles of PCR before being run on 1.5% agarose gels alongside the appropriate base pair ladder (Hyperladder IV – Bioline, London, UK).

We used two sets of oligonucleotides to assay TRAK2 expression designed against the sequence published by Beck *et al.* 2002 (NCBI Accession Number NM_172406.3). The primer pairs were as follows: TRAK2-A (F) 5′-AATGTGGAGAGAGCGCAGTG-3′ (bases 3298–3318) and (R) 5′- CCAAGGCAAGGAAACGTAGC-3′ (bases 3455–3475 – fragment size 158 base pairs) and TRAK2-B (F) 5′-AAGCCGAGAAGCAGAAATGG-3′ (bases 3012–3032) and (R) 5′- GGTGAGGTTGTGCAAACTGG -3′ (bases 3158–3178 – fragment size 147 base pairs). Primers were re-suspended in nuclease free water, to a concentration of 2.5 μM.

Western blotting was carried out on astrocyte cultures and mouse whole brain, using methods previously described (Bahia *et al.* 2012). To probe for TRAK2 a rabbit antibody raised against residues 8-633 that also recognizes TRAK1 was used (kind gift of Professor F A Stephenson, UCL School of Pharmacy) and glyceraldehyde 3-phosphate dehydrogenase (GAPDH - monoclonal goat anti-mouse, 1 : 10000; Life Technologies) was used as a reference protein. Primary antibodies were incubated overnight at 4°C in tris buffered saline (TTBS) supplemented with Tween-20 (20 mM tris buffer (pH 7.5 – Sigma) containing 0.4% Tween-20 (Sigma) and 1% non-fat milk powder). The blots were washed in TTBS and incubated with the corresponding goat anti-rabbit/goat anti-mouse secondary antibodies conjugated to horseradish peroxidase (1 : 5000; Sigma) at 23°C for 1 h. After washing, membranes were incubated with enhanced chemiluminescence reagent (GE Healthcare, Little Chalfont, UK) for 2 min. The enhanced chemiluminescence reagent was prepared according to the manufacturer's protocol. Blots were imaged using the Chemidoc MP imaging system (Biorad) for chemiluminescence and analysed by the corresponding ImageLab software (Bio-Rad Laboratories, Hercules, CA, USA). Images were exported from the software at a resolution of 600dpi into photoshop CS3 and used for representation.

### Transfection and drug treatments

We used a V5 tagged GLT-1 plasmid, the MAST-KREK isoform corresponding to the predominant splice variant, also known as GLT-1a (Peacey *et al.* 2009), the commercially available pDs Red 1-mito plasmid (Clontech, Mountain View, CA, USA) for mitochondrial tracking and a green fluorescent protein (GFP) tagged dominant negative trafficking adhesion kinase 2 (DN TRAK2) plasmid (Brickley and Stephenson 2011). Primary mouse astrocytes and co-cultures (10 and 18 DIV) were single, double or triple transfected with plasmids using Lipofectamine 2000 (Life Technologies) according to manufacturer's instructions, and as previously described (Peacey *et al.* 2009). This transfection method results in predominant astrocyte transfection. Whilst some neurons in co-cultures were transfected; they were excluded from analysis based on cell body size and morphology. Cells were left for 48 h before washing and fixing. After transfection, cells were washed with PBS and fresh media was replaced. Transfected cells were then treated with vehicle (0.1% sterile PBS) or the Rho Kinase inhibitor Y27632 (100 μM, Merck-Millipore, Watford, UK) for 24 or 48 h. In some experiments, co-cultures were further treated with KCl (15 mM, pH 7.4) for 1 h following vehicle or Y27632 administration. In additional experiments co-cultures were treated with ouabain (1 μM), gramicidin (10.6 μM) or monensin (20 μM) for 4 h before fixing.

### Immunofluorescence and cell imaging

Following treatments, cells were fixed in 4% paraformaldehyde in PBS (30 min). Unless otherwise stated, following fixation, cells were blocked and permeabilized in 1% normal goat serum and 0.2% Triton-X100 (Sigma) in PBS, for 1 h at 23°C. For visualization of V5-GLT-1, cells were incubated overnight at 4°C with a mouse monoclonal anti-V5 primary antibody (R960-25; Life Technologies) diluted 1 in 1000 in PBS containing 1% Normal goat serum. Coverslips were washed three times for 5 min in PBS then incubated with a goat anti-mouse secondary antibody conjugated to Alexafluor 488 (Life technologies) for 90 min, at 23°C. Coverslips were then washed with PBS, with some incubated for 5 min with Hoescht 33342 in PBS (4 μg/mL; Life Technologies) before mounting on slides using Vectashield mounting medium (Vector Laboratories, Burlingame, CA, USA). Images were collected on an (Zeiss Axioskop 2; Carl Zeiss Microscopy, LLC, Thornwood, NY, USA) upright microscope using 40x and 100x Plan-NeoFluar objectives, collected using a colour camera (Zeiss AxioCam HRc colour camera, Carl Zeiss Microscopy) and processed using Axiovision Software (Carl Zeiss Microscopy). Zeiss Filter Sets 02/10 and 15 for DAPI/FITC/Rhodamine were used to visualize staining. High-resolution images were created using Photoshop CS3. Confocal z-stacks were generated using an inverted Leica DMIRE2 microscope and processed using the accompanying Leica confocal software (Manheim, Germany). A Leica HCX PL APO 63x objective was used to generate 1024 × 1024 high-resolution z-stacks. Representative *z*-stacks were opened in ImageJ (LOCI plugin – University of Wisconsin-Madison, Madison, WI, USA) and deconvolved using the Tikhonov–Miller algorithm (Deconvolution Lab plugin – Biomedical Image Group, EPFL, Lausanne, Switzerland) for clarity before being exported to Photoshop CS3. 3D isosurface and fluorescence plots were derived through Volocity software (6.1.1 Edition, Perkin Elmer, Cambridge, UK). For the data shown in Table1, a total of 20 z-stacks were collected and analysed from three independent cultures, using data from seven transfected cells from two cultures and six transfected cells from the third and combined the results for statistical analysis, with the exception of DN TRAK2 transfected pure astrocyte cultures where 10 z-stacks were collected from three independent cultures (3–4 cells from each culture).

**Table 1:** Quantification of mitochondrial: GLT-1 co-localization in cultures analysed by confocal microscopy

	Astrocytes alone	Astrocytes cultured with neurons	Astrocytes alone + DNTRAK2	Astrocytes cultured with neurons + DNTRAK2
No drug treatment	0.04 ± 0.04 (20)	0.19 ± 0.03 (20)**	0.06 ± 0.06 (10)	0.18 ± 0.02 (20)*
Y27632	0.05 ± 0.04 (20)	−0.05 ± 0.04 (20)	0.09 ± 0.03 (20)	0.05 ± 0.03 (20)

Primary cultures were co-transfected with V5-GLT-1 and pDs-Red1 mito (with or without dominant negative TRAK2) then treated with vehicle or Y27632 (100 μM, 24 h) and analysed for GLT-1: mitochondrial co-localization. Values represent Costes Pearson's correlation coefficient (−1 to +1; strong negative to strong positive correlation) with SEM and number of cells analysed for each condition. *n* = 3 independent cultures. Data analysed by Two-Way anova. ND = not determined. Asterisks represent significance when compared with astrocytes alone (^*^^*^) and astrocytes plus DN TRAK2(^*^).

### Co-localization analysis

Co-localization was determined using confocal microscopy and Volocity image analysis software. Firstly, a library of files was generated by importing all the TIFF images into the software. Using the green and red 8-bit images (V5 and pDs Red-1 Mito respectively), image sequences were generated allowing co-localization in the whole image to be determined using automatic thresholding, to generate the best threshold for each image sequence (Costes *et al.* 2004). The software allowed Costes Pearson's correlation coefficient (−1 to +1) and correlation coefficient M_1_ and M_2_ (representing co-localized voxels in each channel) to be determined. We analysed 20 images per parameter (minimum of 50 frames per channel) and then averaged the Costes Pearson's correlation coefficient to determine the degree of co-localization for each experimental condition.

### Statistical analysis

Statistical comparison of treatment groups was carried out using anova followed by Dunnett's *post hoc* test (Prism, GraphPad Software Inc., La Jolla, CA, USA).

## Results

### Mitochondrial and GLT-1 co-localization in astrocytes

**Fig 1:**
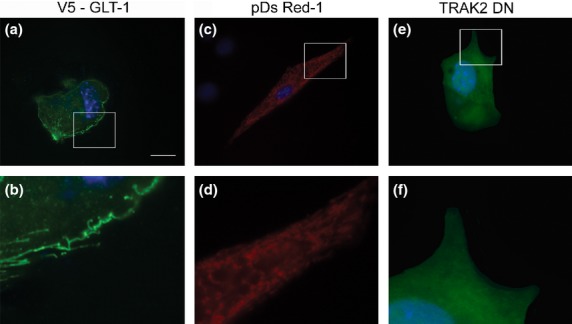
Expression of transfected proteins in primary mouse astrocytes. (a, b) Immunohistochemistry showing V5 expression (green) to localize Glutamate Transporter 1 (GLT-1). (c, d) pDs Red-1 mito expression to localize astrocyte mitochondria. (e, f) Green fluorescent protein (GFP) expression following Dominant negative (DN) TRAK2 transfection. (b, d, f) represent higher magnifications of the parts of images boxed in (a, c, e). Scale bar (a, c, e) = 10 μm (b, d, f) = 2 μm.

The V5 tagged GLT-1 (V5-GLT-1) plasmid results in predominant expression of GLT-1 at the plasma membrane, with negligible expression in intracellular compartments, as previously shown (Peacey *et al.* 2009) (Fig.1a and b). Transfection with pDs Red-1 mito, a mitochondrial tracking plasmid shows discrete punctate distribution throughout the cytosol of astrocytes, with little localization of mitochondria in filopodia (Fig.1c and d). The construct expressing the dominant negative trafficking kinesin 2 (DN TRAK2, previously characterized and described by Brickley and Stephenson (2011)) also expresses GFP through an internal ribosome entry site motif. GFP was evenly distributed throughout the cytoplasm of transfected astrocytes (Fig.1e and f).

**Fig 2:**
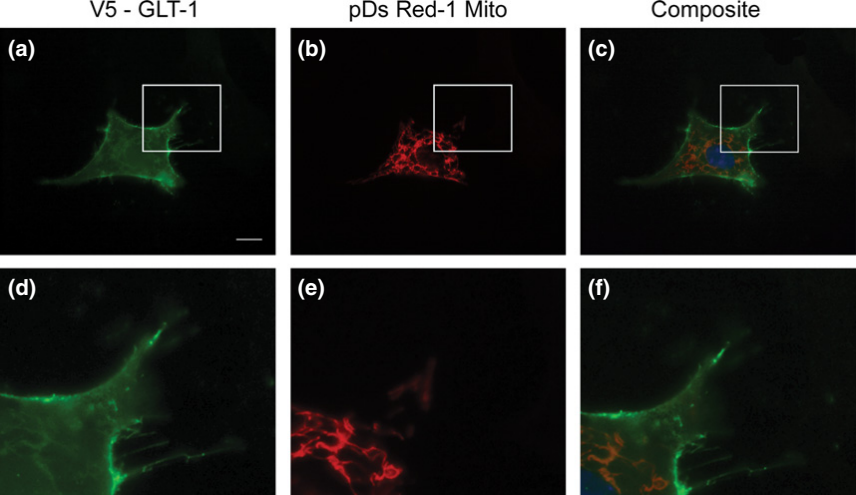
Limited co-localization of mitochondria with GLT-1 in primary astrocytes. Fluorescence microscopy of primary mouse astrocytes transfected with plasmids encoding V5-GLT-1 and DsRed-1 mito. (a, b) show staining with anti-V5 (green), (c, d) show mitochondria (red) and (e, f) show a composite image allowing visual analysis of co-localization. Nuclei are counterstained with Hoescht 33342 (blue). (b, d, f) represent higher magnifications of the parts of images boxed in (a, c, e). Scale bar (a, c, e) = 10 μm, (b, d, f) = 1 μm.

To explore potential links between mitochondria and GLT-1, firstly astrocytes were double transfected with pDs red-1 mito and V5-GLT-1 plasmids (Fig.2) and analysed by confocal microscopy. The overlap between green (GLT-1) and red (mitochondria) fluorescence was assessed by eye and using Volocity software. Very few areas of co-localization were visible by eye, an observation further reinforced by a Costes Pearson's coefficient of 0.04 ± 0.04 (Table1) indicating no correlation between V5-GLT-1 and mitochondria in primary cultures of pure astrocytes. Using unpermeablized cultures, to visualize cell-surface GLT-1 exclusively gave the same result. We also found a negative correlation between both proteins (−0.06 ± 0.04) when V5-GLT-1 and pDs Red-1 mito are co-expressed in COS-7 cells.

### Y27632 toxicity in primary cultures

To show that astrocyte stellation was not because of toxicity, astrocytes were treated with varying doses of the inhibitor Y27632 (1, 10 and 100 μM). At 24 h, each concentration showed no significant difference in MTT turnover comparative to saline treated controls, as determined by one-way anova followed by Dunnett's *post hoc* to compare all columns (*F* = 0.97, *p* = 0.4531), data not shown. Phase contrast time lapse videos taken over 24 h (Data S1) also support this finding. In co-cultures, we stained for MAP2 and GFAP after 4 h and 24 h Y27632 treatment. Co-cultures showed no visible difference in neuronal number or morphology upon rho kinase inhibition at both time points (data not shown).

### Effect of Rho kinase inhibition and co-culture with neurons on GLT-1: mitochondrial co-localization

**Fig 3:**
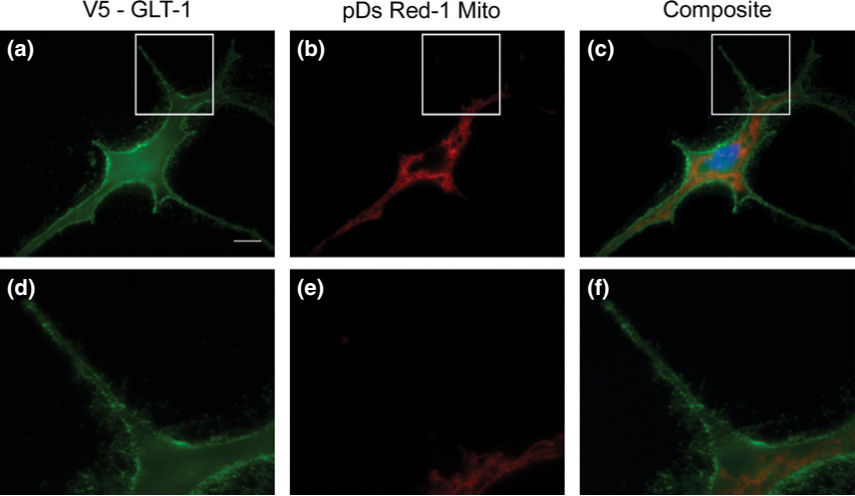
Rho kinase inhibition causes astrocyte stellation without enhancing co-localization of mitochondria and GLT-1 in pure astrocyte cultures. Astrocytes co-transfected to express V5-GLT-1 (green) and DsRed-1 mito (red), were treated with Y27632 (100 μM, 24 h). (a, b) show staining with anti-V5 (green), (c, d) show mitochondria (red) and (e, f) show a composite image allowing visual analysis of co-localization. Nuclei are counterstained with Hoescht 33342 (blue). (b, d, f) represent higher magnifications of the parts of images boxed in (a, c, e). Scale bar (a, c, e) = 10 μm, (b, d, f) = 1 μm.

As Rho kinase inhibition increases astrocyte stellation and increases GLT-1 expression at the cell surface (Lau *et al.* 2011), we hypothesized it would increase co-localization of mitochondria with GLT-1. Therefore, we treated double transfected cells with the Rho Kinase inhibitor Y27632 (100 μM) for 24 h then monitored mitochondrial and V5-GLT-1 co-localization (Fig.3). Co-cultures were treated for 4 h with the inhibitor. Rho Kinase inhibition caused marked astrocyte stellation and formation of many fine filopodia, decorated with V5-GLT1 (Data S2) but did not markedly alter the distribution of mitochondria within the astrocytes. Fewer GLT-1 co-localized mitochondria were visible by eye and as for control cultures, in Y27632 treated cultures, mitochondria and GLT-1 showed no positive correlation (Costes Pearson's coefficient of 0.05 ± 0.04, Table1).

### Neurons drive co-localization of GLT-1 and mitochondria

**Fig 4:**
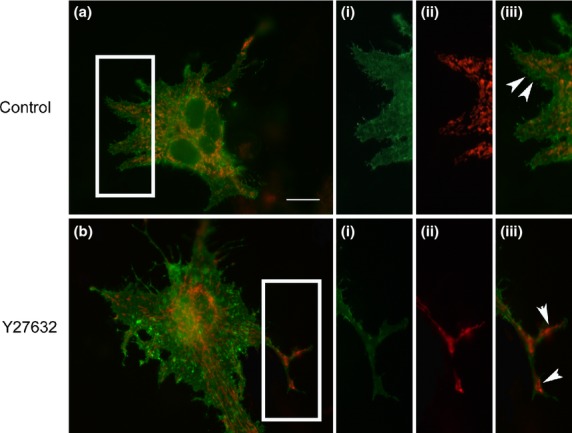
Glutamate Transporter 1 (GLT-1): mitochondria co-localization in astrocytes co-cultured with neurons. Astrocyte, neuron co-cultures were co-transfected with plasmids encoding V5-GLT-1 (green) and DsRed-1 mito (red). (a) Representative untreated control cell and (b) representative cell treated with Y27632, each with higher magnification insets showing (i) V5-GLT-1, (ii) mitochondria (iii) overlay of both revealing increased mitochondrial localization in filopodia, but with limited GLT-1: mitochondrial co-localization in Y27632 treated astrocytes. White arrowheads indicate the appearance of mitochondria in astrocyte filopodia when cultured with neurons (a(iii)) and when co-cultured astrocytes change to a stellate morphology (b(iii)). Scale bar (a, b) = 10 μm, (i)–(iii) = 5 μm.

**Fig 5:**
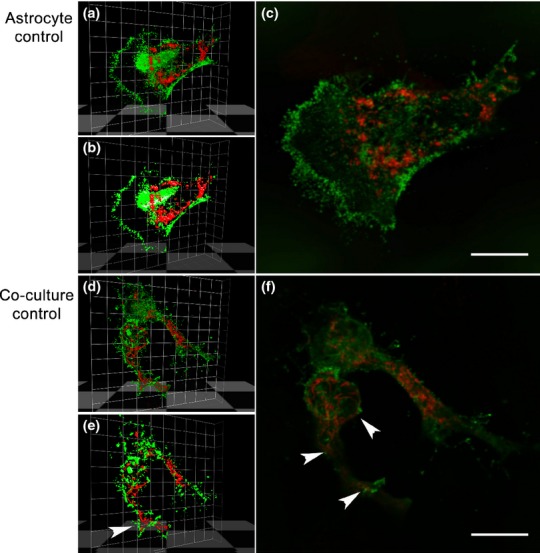
Co-localization in co-cultures using confocal microscopy. Astrocytes in pure cultures (a–c) have fewer co-localized voxels in comparison to co-cultures (d–f) as determined by Volocity software. (a+d) Fluorescence 3D plots showing co-localization of green and red pixels in the z-stacks. (b+e) 3D iso-surface reconstruction showing co-localized points in each z-stack. Green channels represent V5-GLT-1 fluorescence, whereas pDs Red-1 mito expression can be seen in the red channel. c+f) Co-localization observed in deconvolved z-stacks (Tikhonov-Miller algorithm). After deconvolution, a snapshot was taken from the z-stack and processed using photoshop CS3. White arrowheads indicate co-localized voxels in astrocytes cultured with neurons. Mitochondria can be found throughout the cell body and processes. Scale bar = 10 μm.

Next, astrocyte-neuron co-cultures were generated as astrocytes in the presence of neurons would be expected to have mitochondria and GLT-1 more densely packed into astrocyte processes and possibly filopodia, enhancing co-localization. Cultures were transfected with plasmids encoding V5-GLT-1 and DsRed-1-mito and analysed with and without treatment with Y27632. Under these conditions, there was increased recruitment of mitochondria into filopodia (Fig.4a), which was particularly pronounced following Rho kinase inhibition (Fig.4b). While, in co-cultures, there was modest overlap of mitochondria and GLT-1 observed by eye, quantification revealed significant positive correlation with a Costes Pearson's coefficient of 0.19 ± 0.03, (Table1). Two-Way anova revealed a significant increase in co-localization between GLT-1 and mitochondria in co-culture conditions when compared with pure astrocyte controls (*F*_(1,20)_ = 11.8, *p* < 0.001). Representative three-dimensional fluorescence and isosurface plots are shown in Figure5.

In astrocytes co-cultured with neurons and treated with Y27632, while there appeared to be increased distribution of mitochondria in astrocyte processes (Fig.4b) this treatment significantly decreased mitochondrial and V5-GLT-1 co-localization as the Costes Pearson's coefficient was −0.05 ± 0.04 (Table1). The decrease in co-localized GLT-1 and mitochondria caused in astrocytes co-cultured with neurons stimulated with Y27632 was highly significant (*F*_(1,20)_ = 10.45 *p* < 0.01).

### Effect of neuronal stimulation and sodium ion gradient manipulation on GLT-1/mitochondrial co-localization

Whilst we showed that there is indeed a positive correlation between GLT-1 and mitochondria when neurons are cultured with astrocytes, we hypothesized that stimulating neuronal depolarization would encourage further re-distribution of mitochondria and GLT-1 to astrocyte processes, enhancing co-localization. We stimulated co-cultures (18 DIV) with 15 mM KCl for 1 h and analysed co-localization. Interestingly, we found no further increase in co-localization in co-culture controls treated with KCl (0.16 ± 0.05), suggesting that the degree of co-localization could not be further stimulated.

We also carried out a series of experiments to test the effect of manipulating the sodium ion gradient in co-cultures. Astrocytes have numerous mechanisms which regulate sodium ions and glutamate transport is dependent on the sodium gradient (Rose and Karus 2013). We used ouabain (1 μM), a Na/K-ATPase inhibitor also reported to increase co-localization of Na/K-ATPase to GLT-1 (Rose *et al.* 2009), gramicidin (10.6 μM), a sodium ionophore also reported to inhibit Na/K-ATPase (Takada *et al.* 2008) and monensin (20 μM), an alternative sodium ionophore, that is also reported to stimulate Na/K-ATPase in astrocytes (Sheean *et al.* 2013). None of these agents had a significant effect on the extent of co-localization of mitochondria with GLT-1 (results not shown).

### Effect of TRAK2 inactivation on astrocyte morphology, mitochondrial distribution and GLT-1: mitochondria co-localization

**Fig 6:**
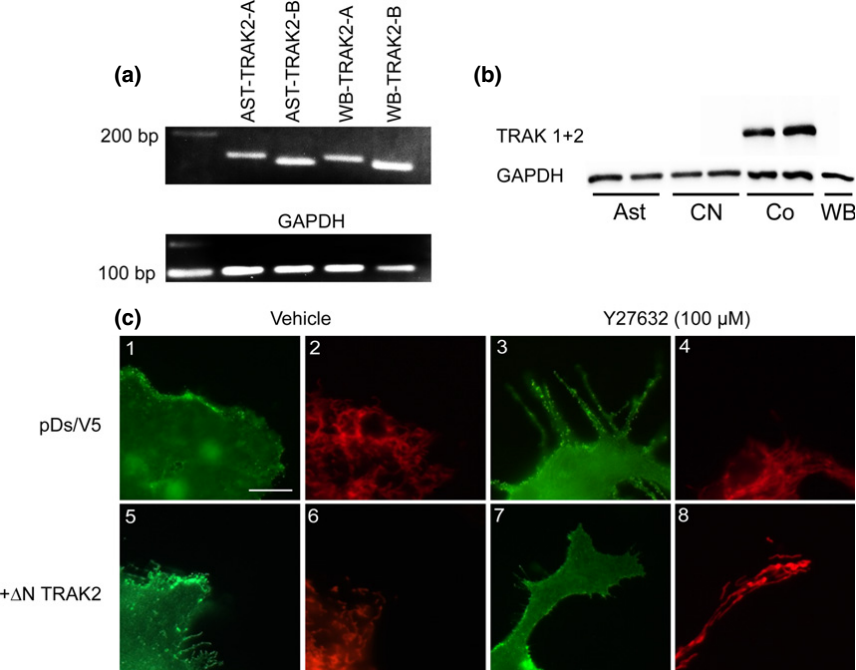
Dominant negative TRAK2 does not alter mitochondrial: Glutamate Transporter 1 (GLT-1) co-localization in astrocytes. (a)TRAK2 mRNA is present in pure astrocyte cultures (AST) and whole brain (WB), as identified using two distinct pairs of oligonucleotides TRAK2-A (158 bp fragment) and TRAK2-B (147 bp fragment). GAPDH was used as a housekeeping gene; to show equal concentrations of cDNA were loaded. (b) TRAK2 (102 kDa) protein is expressed in co-cultures (Co) but undetectable in pure cultures of cortical astrocytes (Ast), neurons (CN) and whole brain (WB). GAPDH was used as a reference protein for protein loading. (c) Immunofluorescence microscopy showing GLT-1 (green) and mitochondria (red) in astrocyte-neuron co-cultures transfected with plasmids encoding DsRed-1 mito and V5-GLT-1 (1-4), with DNTRAK2 (5-8). Cells were treated with vehicle (1,2,5,6) or the rho kinase inhibitor Y27632 (3,4,7,8). Scale bar (a–h) = 5 μm.

Using PCR we found that under normal conditions, astrocytes express TRAK2 mRNA (Fig.6a). Interestingly, using Western Blotting, TRAK2 protein was not detectable in astrocytes cultured alone, but prominent in astrocytes co-cultured with neurons (Fig.6b). We tested the effect of co-expression of a dominant negative form of TRAK2 (DN TRAK2) on co-localization of GLT-1 and mitochondria in primary astrocytes. We measured co-localization in the presence and absence of the rho kinase inhibitor, Y27632 in pure cultures (not shown) and astrocytes co-cultured with neurons (Fig.6). We note that expression of DN TRAK2 had no obvious effect on astrocyte morphology, nor did it markedly alter the distribution of mitochondria within astrocytes, either in pure cultures or when cultured with astrocytes. As for the previous experiments, very little co-localization was observable by eye. Measurement of co-localization using Volocity software (Table1) revealed that co-localization of GLT-1 and mitochondria was similar to values found in cultures without DN TRAK2 expression. Two-Way anova revealed no significant effect on co-localization between GLT-1 and mitochondria caused by either Y27632 treatment (*F*_(1,20)_ = 2.29, *p* = 0.135) or co-culturing with neurons (*F*_(1,20)_ = 2.07, *p* = 0.155).

## Discussion

Recent evidence has pointed to the existence of a physical link between mitochondria and GLT-1 in astrocytes (Genda *et al.* 2011). While this link has been convincingly demonstrated biochemically, there is so far only a single detailed examination of the overlap using imaging techniques (Jackson *et al.* 2014). To examine this interaction in more depth we used a model system of primary mouse astrocytes and astrocyte neuron co-cultures to monitor levels of co-localization between epitope tagged GLT-1 (Peacey *et al.* 2009) and a mitochondrial tracking marker (DS Red 1 mito).

We used confocal microscopy to investigate the association between GLT-1 and mitochondria using Volocity software to calculate the level of co-localization between these two proteins. We found no association between GLT-1 and mitochondria in pure primary astrocytes, whereas in primary astrocytes cultured with neurons, there was an increased distribution of mitochondria into the thicker processes, as well as a small meaningful positive correlation between GLT-1 and mitochondria, that is a greater degree of overlap than expected by chance. Volocity software provides a sophisticated and useful method of analysing co-localization through the generation of three-dimensional fluorescence/isosurface maps to identify true regions of co-localization. The 3D data show more co-localized voxels in control cells from the co-cultures in comparison to control cells in astrocyte cultures. Altogether our results suggest that in co-cultures, that is cultures containing functional neurons; mitochondria invade filopodia and become closer to the plasma membrane, with some co-localizing with GLT-1. The significant increase in correlation between mitochondria and GLT-1 localization in co-cultures compared to pure astrocyte cultures is consistent with the notion that neuronal activity drives GLT-1 mitochondrial co-localization. This observation supports the data of the Robinson group, who surveyed GLT-1:mito co-localization in astrocytes in brain slices, although they observed a greater degree of co-localization than we find in primary astrocyte cultures (Genda *et al.* 2011; Jackson *et al.* 2014). We note that whilst co-localization is present in our experiments, the proportion of mitochondria co-localizing with GLT-1, and vice versa, is very small, we estimate < 1%, even under conditions of co-culture. Therefore, the physiological significance of this small degree of co-localization is still questionable, particularly as there is little evidence of mitochondria being present in the fine astrocyte filopodia which are rich in glutamate transporters (Hertz *et al.* 2007). We note that the diameter of the smallest processes (peripheral astrocyte processes) are reported to be < 50 nm, too small to house mitochondria (Lavialle *et al.* 2011).

Stimulation of co-cultures with 15 mM K^+^ does not further enhance co-localization between these two proteins (0.16 ± 0.05), suggesting that co-culture alone permits the maximum level of co-localization. We also show that the extent of co-localization is not affected by manipulation of the sodium ion gradient or modulation of Na/K ATPase. Further studies will provide insight into whether there are conditions which further promote such co-localization.

We note that the method used to culture astrocytes here supports high levels of L-glutamate uptake (Tortarolo *et al.* 2004; Peacey *et al.* 2009). We have chosen to use transfection of a tagged GLT-1 construct to enable visualization of the transporter in fine processes, which provides a significant technical advantage above visualization of endogenous GLT-1. The localization and properties of our tagged GLT-1 in astrocytes is indistinguishable from native GLT-1, determined by a range of biochemical methods (Peacey *et al.* 2009), however, we cannot rule out that the tagged GLT-1 used here may differ in its coupling to mitochondria to endogenous GLT-1, accounting for a degree of difference in the proportion of co-localization reported here compared to the two other published reports (Genda *et al.* 2011; Jackson *et al.* 2014).

In pure astrocyte cultures, mitochondrial localization in astrocyte processes was rare. While we did not quantify mitochondrial distribution and movement in this study, we note that two of the treatments used: co-culture with neurons and treatment with the rho kinase inhibitor, Y27632, resulted in mitochondria being readily detectable in the thicker astrocyte processes. These observations support recent data showing that mitochondria in astrocytes are highly motile, with their re-localization mediated by intracellular calcium (Kremneva *et al.* 2013). The movement of mitochondria to astrocyte processes brings them closer to the plasma membrane, so even if there is not direct co-localization there is less distance for diffusion of ATP required for the sodium-potassium ATPase to drive GLT-1 activity. Our current data suggest that neuronal influences alone are sufficient to cause re-localization of mitochondria, and that it is not the sodium ion gradient or Na/K ATPase activity which is the main driver. Further work remains to detail the mechanisms regulating glutamate transporter: mitochondrial interaction.

This study is the first report of expression of TRAK2 mRNA in astrocytes and inhibition of TRAK2 in astrocytes. The currently available reagents precluded a detailed study of TRAK2 localization at the cellular level. Our results suggest an intriguing regulation of TRAK2, with protein expression in astrocyte cultures dependent on the presence of neurons. The functions of TRAK2 in astrocytes are not yet known. In this study, we note that treatment with dominant negative TRAK2 did not markedly affect cell shape, GLT-1 distribution or mitochondrial distribution in either pure astrocyte cultures or astrocyte neuron co-cultures. This preliminary evidence suggests that TRAK2 is not a mediator of GLT-1: mitochondrial interactions in astrocytes.

In closing, here we show that there is no co-localization of GLT-1 and mitochondria in our astrocyte cultures, however, in astrocyte-neuron co-cultures; we begin to see a small positive correlation. This correlation may increase depending on the age of the cell population, which if true, may represent a more biologically significant coupling.

## Abbreviations

CNS: Central nervous system
DIV: Days *in vitro*
DMEM/F-12(HAM): Dulbecco's Modified Eagle Medium: Nutrient Mixture F-12
DN: Dominant negative
EAAT2: Excitatory Amino Acid Transporter 2
EBSS: Earles balanced salt solution
FBS: Foetal bovine serum
GFP: Green fluorescent protein
GLT-1: Glutamate Transporter 1
NGS: Normal goat serum
PBS: Phosphate-buffered saline
ROCK: Rho Kinase
SEM: Standard Error of the Mean
TRAK2: Trafficking kinesin protein 2
Y27632: (1R,4r)-4-((R)-1-aminoethyl)-N-(pyridin-4-yl)cyclohexanecarboxamide

## Supporting Information

Data S1 Phase contrast time lapse video (24 h) showing astrocyte stellation under rho kinase inhibition using Y27632 (100 μM).

Data S2 Confocal z-stack showing double transfected astrocytes (V5-GLT-1 and pDs-Red 1 mito; green and red respectively) treated with Y27632 for 24 h.
