# Supplementary material for: Neuronal influences are necessary to produce mitochondrial co-localization with glutamate transporters in astrocytes
Source: J Neurochem. 2014 Jun 16;130(5):668–77. doi: 10.1111/jnc.12759 (PMC4283053; doi:10.1111/jnc.12759)
Supplement: Data S2 — Confocal z-stack showing double transfected astrocytes (V5-GLT-1 and pDs-Red 1 mito; green and red respectively) treated with Y27632 for 24 h. [file jnc0130-0668-SD3.pdf]

Neuronal influences are necessary to produce mitochondrial co-localization with glutamate transporters in astrocytes

Christopher I Ugbode[*‡], Warren D. Hirst†, Marcus Rattray[‡1]

Supporting Information 1

Astrocyte stellation under rho kinase inhibition.

Phase contrast time lapse video (24 h) showing astrocyte stellation under rho kinase inhibition using Y27632 (100µM). The video starts roughly 40 minutes after the application of the drug due to setting up the microscope; indicating that the morphological change happens rapidly; without affecting cell number (over time).

Supporting information 2.

Peripheral astrocyte processes emanating from filpodia express V5-GLT-1.

Confocal z-stack showing double transfected astrocytes (V5-GLT-1 and pDs-Red 1 mito; green and red respectively) treated with Y27632 for 24 h. The filpodia are decorated with GLT-1 however there is lack of mitochondrial distribution in the finer processes.
